# Adsorption and Release Properties of Drug Delivery System Naproxen-SBA-15: Effect of Surface Polarity, Sodium/Acid Drug Form and *pH*

**DOI:** 10.3390/jfb13040275

**Published:** 2022-12-05

**Authors:** Ľuboš Zauška, Eva Beňová, Martina Urbanová, Jiří Brus, Vladimír Zeleňák, Virginie Hornebecq, Miroslav Almáši

**Affiliations:** 1 Department of Inorganic Chemistry, Faculty of Science, P. J. Šafárik University, Moyzesova 11, SK-041 01 Košice, Slovakia; 2 Department of NMR Spectroscopy, Institute of Macromolecular Chemistry CAS, Heyrovského nám. 2, CZ-162 06 Prague, Czech Republic; 3 Aix Marseille Univ, CNRS, MADIREL, UMR 7246, 13397 Marseille, France

**Keywords:** SBA-15, naproxen sodium/acid, drug delivery system, surface modification, polar and nonpolar functional groups, *pH*

## Abstract

Mesoporous silica SBA-15 was prepared via sol-gel synthesis and functionalized with different types of organosilanes containing various organic functional groups: (3-aminopropyl)triethoxysilane (SBA-15-NH_2_), (3-mercaptopropyl)triethoxysilane (SBA-15-SH), triethoxymethylsilane (SBA-15-CH_3_), triethoxyphenylsilane (SBA-15-Ph), and (3-isocynatopropyl)triethoxysilane (SBA-15-NCO). The prepared materials were investigated as drug delivery systems for naproxen. As model drugs, naproxen acid (HNAP) and its sodium salt (NaNAP) were used. Mentioned medicaments belong to the group of non-steroidal anti-inflammatory drugs (NSAIDs). The prepared materials were characterized by different analytical methods such as transmission electron microscopy (TEM), infrared spectroscopy (IR), nitrogen adsorption/desorption analysis (N_2_), thermogravimetric analysis (TG), ^1^H, ^13^C and ^23^Na solid-state nuclear magnetic resonance spectroscopy (^1^H, ^13^C and ^23^Na ss-NMR). The abovementioned analytical techniques confirmed the successful grafting of functional groups to the SBA-15 surface and the adsorption of drugs after the impregnation process. The BET area values decreased from 927 m^2^ g^−1^ for SBA-15 to 408 m^2^ g^−1^ for SBA-15-NCO. After drug encapsulation, a more significant decrease in surface area was observed due to the filling of pores with drug molecules, while the most significant decrease was observed for the SBA-15-NH_2_ material (115 m^2^ g^−1^ for NaNAP and 101 m^2^ g^−1^ for HNAP). By combining TG and nitrogen adsorption results, the occurrence of functional groups and the affinity of drugs to the carriers’ surface were calculated. The dominant factor was the volume of functional groups and intermolecular interactions. The highest drug affinity values were observed for phenyl and amine-modified materials (SBA-15-Ph = 1.379 μmol m^−2^ mmol^−1^ for NaNAP, 1.761 μmol m^−2^ mmol^−1^ for HNAP and SBA-15-NH_2_ = 1.343 μmol m^−2^ mmol^−1^ for NaNAP, 1.302 μmol m^−2^ mmol^−1^ for HNAP) due to the formation of hydrogen bonds and *π-π* interactions, respectively. Drug release properties and kinetic studies were performed at *t* = 37 °C (normal human body temperature) in different media with *pH* = 2 as simulated human gastric fluid and *pH* = 7.4, which simulated a physiological environment. Determination of drug release quantity was performed with UV-VIS spectroscopy. The surface polarity, *pH* and naproxen form influenced the total released amount of drug. In general, naproxen sodium salt has a higher solubility than its acid form, thus significantly affecting drug release from surface-modified SBA-15 materials. Different *pH* conditions involved surface protonation and formation/disruption of intermolecular interactions, influencing both the release rate and the total released amount of naproxen. Different kinetic models, zero-order, first-order, Higuchi and Hixson–Crowell models, were used to fit the drug release data. According to the obtained experimental results, the drug release rates and mechanisms were determined.

## 1. Introduction

Infectious diseases follow the human body during the entire phylogenetic and ontogenetic evolution. In human history, there were diseases with high-rate mortality, e.g., bubonic plague, influenza, and smallpox, and the simultaneous worldwide spread of COVID-19 caused by the coronavirus SARS-CoV-2, cannot be overlooked [1,2]. Infectious disease incidence has brought enormous health, social and economic problems [3]. Infectious diseases caused by viruses usually lead to inflammation in the human body, specifically in organs, bones, blood, etcetera. Inflammation is a natural immune response to infection, but there is a high risk where inflammation triggers both cardiovascular disease [4] and ischemic stroke [5], which can be observed with COVID-19 infection.

The biochemical mechanism of inflammation is a very simplified means of interleukin (IL)-1b production via cells as the response to the bacterial or viral presence in the intracellular environment. (IL)-1b is a “starter” for inflammation mediator production as COX-2 (COX—cyclooxygenase) [6]. COX-2 with COX-1 induces arachidonic acid production, where this acid is transformed into prostaglandin endoperoxide H_2_(PGH_2_), which starts inflammation in the human body [7]. NSAIDs (non-steroidal anti-inflammatory drugs), such as naproxen, work as reverse inhibitors for COX-1 and COX-2. The result of described inhibition is blocking prostaglandin production in human cells [8].

There is a possibility of treating the inflammation with NSAID that is loaded into drug delivery systems (DDSs), such as metal-organic frameworks [9,10,11], mesoporous silica [12,13,14] and organic polymers [15,16,17]. In biotechnology research, many other types of materials are used for drug delivery systems [18]. The significant advantages of these materials lie in their post-synthetical chemical modification, chemical stability in the physiological environment and biocompatibility. Mesoporous silicas are inorganic compounds widely studied as DDSs for their versatility and simple surface modification. These properties make mesoporous silica a great candidate as DDS for anticancer therapy, wound healing, vitamin transport and inflammation treatment.

In the literature, research papers focus on the surface functionalization of mesoporous silica nanoparticles using various organic and inorganic compounds [19,20], where each modified material has unique chemical and physical properties. The aminopropyl functional group is a frequently used molecule in grafting for the preparation of DDS. For example, works [21,22,23] focus on studies concerning amino-functionalized mesoporous silica surfaces used to store drugs. These studies showed that amine modification influences the loading capacity of drugs such as aspirin and ibuprofen due to intermolecular interactions. Furthermore, the drug release is influenced by the total amount of amine functional groups at the surface. Other examples concern polymers or macromolecules that have been used to modify the surface to affect the drug release rate from silica-based DDS [24,25]. Studies have shown that poly(*ε*–caprolactone) had better efficiency for the drug loading of ammonium glycyrrhizinate and sustained release from the prepared matrix. Polyamido(amine) (PAMAM) with hollow silica nanoparticles combination has proven to be suitable for the active delivery system of aluminum phthalocyanine tetrasulfonate for breast cancer therapy. An example of a post-synthetic modification with an inorganic compound is hydroxyapatite for bone therapy using clindamycin as a drug [19]. 

It is well-known that NSAID drugs are widely used for inflammation treatment, but they cause stomach ulcers (after oral administration) in long-term use. Our previous study has shown that the drug delivery system we prepared is suitable for the passive transport of NSAID (diclofenac sodium) in the human body, which can reduce the risk of stomach disease by slow-releasing drugs from pores. Material SBA-15 was modified with branched polyethyleneimines (PEI) as thermosensitive gatekeepers that are open at higher temperatures (42 °C) caused by fever [26]. Other intelligent systems we also focused on were light-sensitive DDSs composed of surface-modified mesoporous silica with coumarin [12,27] and cinnamic acid [28] derivatives that could store naproxen effectively. With the action of visible light, the ligands present on the matrices’ surface undergo photodimerization reactions, and the pores are closed. The irradiation of the materials with UV light leads to pores opening, causing drug release. Other intelligent systems concerned *pH*-sensitive systems composed of *N*-propylaniline and *β*-cyclodextrin (*β*-CD) [29,30] for the targeted treatment of cancer. It is known that the *pH* of the tumor cell environment is lower compared to healthy cells, and therefore *pH*-sensitive systems are intensively developed and studied [31,32,33]. The anticancer drugs 5-fluorouracil and the anti-inflammatory drug naproxen were stored in the thus-prepared carrier, and the pores of the material were subsequently blocked with bulky *β*-CD molecules. *β*-CDs bound to the amine groups on the material’s surface via intermolecular interactions, and their volume prevented drug release. The removal of *β*-CD molecules from the surface of the carrier occurred at reduced *pH* (5–5.5, cancer cell environment), during which the amine groups protonated, thereby removing the *β*-CD molecules and subsequently releasing drugs.

In the present study, mesoporous silica SBA-15 functionalized with different types of polar/nonpolar organosilanes with various functional groups, such as (3-aminopropyl)triethoxysilane (SBA-15-NH_2_), (3-mercaptopropyl)triethoxysilane (SBA-15-SH), triethoxymethylsilane (SBA-15-CH_3_), triethoxyphenylsilane (SBA-15-Ph), and (3-isocynatopropyl)triethoxysilane (SBA-15-NCO), were prepared and their drug adsorption/release properties compared with non-modified silica (SBA-15) were studied. As model NSAID drugs, naproxen acid (HNAP) and sodium salt (NaNAP) were used. Drug release properties and kinetics studies were performed at *t* = 37 °C in different media, with *pH* = 2 representing simulated human gastric fluid and *pH* = 7.4 as simulated body fluid. The obtained results were subsequently fitted by various kinetic models to determine the drug release rate and its mechanism. The article also contains a discussion about interactions between surface modification and naproxen.

## 2. Materials and Methods

The chemicals used in the synthesis of SBA-15 and subsequent post-synthetic modification were purchased from Sigma-Aldrich (Saint-Louis, Missouri, USA) and Across Organics (New Jersey, USA) in the highest available purity.

### 2.1. Synthesis of SBA-15

SBA-15 was prepared by the sol-gel synthesis in which Pluronic 123 (8.5 g) was dissolved in water (70 cm^3^) and 2M HCl (270 cm^3^) in a plastic flask at 35 °C. Subsequently, 20 cm^3^ of triethoxysilane was added to the solution, and the reaction system was stirred at 35 °C for 24 h. After this time, the reaction mixture was transferred to an oven at 90 °C for 24 h. The resulting product, a white powder, was filtered off, washed with 2M HCl, then water, and dried in an oven at 90 °C overnight (yield 11.8 g). The template (Pluronic 123) was removed by a thermolysis process in an oven at 600 °C for 12 h with a heating rate of 0.5 °C min^−1^ (yield 5.6 g).

### 2.2. Post-Synthetic Modification

The post-synthetic surface modification of SBA-15 was performed by the grafting process. Generally, 1 g of SBA-15 was dispersed in 50 cm^3^ of dry toluene, and 25 mmol of respective trialkyloxysilane was added. The reaction system was refluxed for 12 h under an inert nitrogen atmosphere. After reaction completion, the resulting products were filtered, washed with 2M HCl, water and ethanol, and dried in an oven at 60 °C. The weight of the prepared products was in the range of 1.15–1.35 g. The modified products were designated as SBA-15-X (X = NH_2_, NCO, SH, Ph and CH_3_, amino-, isocyanate-, thiol-, phenyl- and methyl-modified materials, respectively).

### 2.3. Drug Loading

Before drug encapsulation in the carriers, porous materials were activated in an oven at 100 °C for 1 h. Then, the drug adsorption (NaNAP/HNAP) was performed by the impregnation method: 200 mg of each unmodified/modified material was dispersed in a 3 cm^3^ aqueous NaNAP solution containing 400 mg of dissolved naproxen sodium. HNAP encapsulation was performed similarly: 200 mg of a matrix was dispersed in a 7 cm^3^ chloroform solution of HNAP in which 400 mg of HNAP was dissolved. The drug loading process was performed at 30 °C using a rotator with a stirring speed of 20 rpm for 24 h. The samples were then filtered and washed with a small amount of water (NaNAP samples) or chloroform (HNAP samples) and dried in an oven at 40 °C overnight. The materials were designated as SBA-15-X-NaNAP for naproxen sodium-loaded samples and SBA-15-X-HNAP for naproxen acid-containing samples (X = no group, NH_2_, NCO, SH, Ph and CH_3_ for pure SBA-15, aminopropyl-, isocyanatepropyl-, thiolpropyl-, phenyl- and methyl-modified samples, respectively). The weights of the prepared dried materials after drug adsorption were: 311 mg for SBA-15-NaNAP, 277 mg for SBA-15-NH_2_-NaNAP, 262 mg for SBA-15-NCO-NaNAP, 273 mg for SBA-15-SH-NaNAP, 231 mg for SBA-15-CH_3_-NaNAP, 290 mg for SBA-15-Ph-NaNAP and 249 mg for SBA-15-HNAP, 260 mg for SBA-15-NH_2_-HNAP, 232 mg for SBA-15-NCO-HNAP, 238 mg for SBA-15-SH-HNAP, 225 mg for SBA-15-CH_3_-HNAP, and 260 mg for SBA-15-Ph-HNAP. As the samples showed an increased weight compared to the matrix used before drug adsorption (200 mg), the obtained results indicate encapsulation of the selected drugs in prepared carriers. 

### 2.4. Drug Release

Samples with encapsulated naproxen (HNAP and NaNAP, 10 mg) were packed into the semipermeable membrane VISKING^®^. Subsequently, the prepared samples were inserted in 100 mL plastic bottles filled with saline solution with different *pH* (2 or 7.4). Mixtures were gently stirred with a magnetic stirrer in a preheated oven at *t* = 37 °C. The solution samples were analyzed in different time intervals: 0.5, 1.5, 3.5, 5.5, 7.5, 9.5, 24, 28, 32, and 48 h. The total amount of released naproxen was determined using UV-VIS spectroscopy at 231 nm, and all experiments were performed in triplicates. Calibration curves were constructed before determining the concentration of released naproxen using HNAP, and NaNAP standards. The correlation coefficients were r^2^ = 0.9986 and r^2^ = 0.9995 for HNAP and NaNAP, respectively (see Figure S1 in ESI).

### 2.5. Characterization

Infrared spectra of prepared materials were measured by FT-IR spectrometer IRTracer-100 by Shimadzu co. using the KBr technique in the wavenumber range of 4000–400 cm^−1^. Samples were mixed with dry KBr (preheated at 600 °C) in a mass ratio 100:1. The measuring settings were 32 scans for a single spectrum with 4 cm^−1^ resolution and sqr–triangle apodization.

TEM images were obtained using a microscope JEOL 2000FX. The samples were finely ground and then suspended in ethanol. The suspension was added dropwise onto a carbon grid and air-dried overnight.

Thermogravimetric (TG) analysis was carried out on a TGA Q500 apparatus with a sample weight of ~20 mg using Pt crucibles. The samples were heated in a dynamic air atmosphere with a flow rate of 60 cm^3^ min^−1^ in a temperature range of 25–800 °C. All measured TG curves were normalized to 100 °C to avoid the effect of solvents on the respective weight losses corresponding to organic molecules (surface-modified groups and drugs).

The textural properties (surface area, pore volume and size) of the prepared materials were determined by nitrogen adsorption/desorption measurements at −196 °C on an ASAP 2020 Micromeritics instrument. Before analysis, samples were degassed and heated under vacuum (150 °C for SBA-15 and 80 °C for modified materials and drug-loaded samples) for 12 h to remove solvent molecules in the cavities. N_2_ adsorption measurements were performed in the range of relative pressures *p/p*_0_ = 0.0002 to 1, through which the respective adsorption/desorption isotherms of the materials were obtained. The surface area of the materials was calculated by the BET (Brunauer–Emmet–Teller) method in the range of relative pressures *p/p*_0_ = 0.05 to 0.2. The volume and pore size were calculated by the BJH (Barrett–Joyner–Halenda) method from the desorption branch of the corresponding nitrogen isotherm.

Solid-state NMR spectra were measured at 11.7 T using a Bruker Avance III HD 500 WB NMR spectrometer (Karlsruhe, Germany, 2013) with a 3.2 mm probe head in ZrO_2_ rotors. The ^13^C MAS and CP/MAS NMR spectra employing cross-polarization (CP) were acquired using the standard pulse scheme at a spinning frequency of 18 kHz. The optimized recycling delay was 2 s, and the cross-polarization contact time was 2 ms. The strength of the spin-locking fields had a *B*_1_(^13^C) field nutation frequency of 62.5 kHz. The number of scans was 10–20 k. Single-pulse ^23^Na MAS NMR spectra were acquired at a spinning frequency of 18 kHz with a pulse width of 1.0 μs (30° flip angle); excitation field intensity *B*_1_(^23^Na) of 83.3 kHz; recycle delays of 2 s; and the number of scans 256–1024 depending on the signal-to-noise ratio. ^1^H MAS NMR spectra were acquired using the standard pulse scheme with a repetition delay of 4 s, and a number of scans of 4. Frictional heating [34] of the spinning samples was compensated by active cooling, and the temperature calibration was performed with Pb(NO_3_)_2_.

UV-VIS spectroscopy measurements were performed in a liquid phase on a Specord 250 (Analytic Jena, Jena, Germany) spectrophotometer in the 200–300 nm wavelength range to determine the drug released amount from prepared DDSs.

## 3. Results and Discussion

### 3.1. Transmission Electron Microscopy

Figure S2 in ESI shows transmission electron microscopy (TEM) images of prepared unmodified SBA-15 particles. As can be seen from Figure S2a, particles have a typical oval shape with a size of 1.6 × 2.5 μm, in which one-dimensional mesopores with hexagonal symmetry (p6mm) with a diameter of about 7 nm are present. The honeycomb-like pores are hexagonally arranged relative to each other, which is confirmed by TEM images taken perpendicularly (see Figure S2b) and along (see Figure S2c) the direction of the pores. Based on our previous experiences [26,27,28,29,30], it was found that the grafting process does not affect the morphology of the particles, and for this reason, TEM measurements of the modified materials were not carried out.

### 3.2. Infrared Spectroscopy

FT-IR spectroscopy was used to qualitatively analyze pure SBA-15, surface-modified materials with different functional groups and drug-loaded samples.

**Figure 1:**
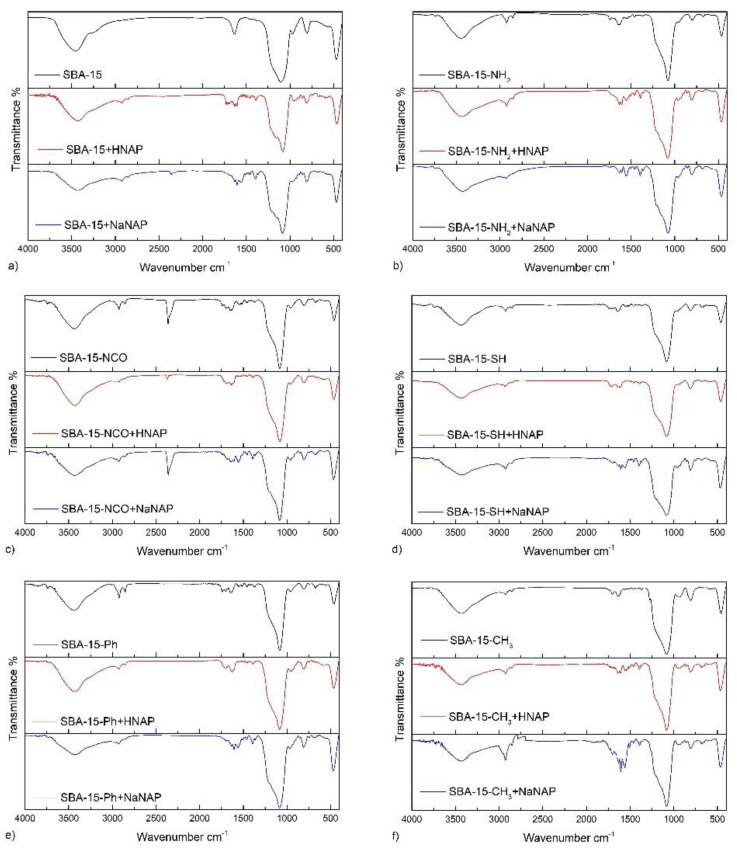
Infrared spectra of surface unmodified/modified materials with encapsulated drug (HNAP/NaNAP) for (**a**) SBA-15, (**b**) SBA-15-NH_2_, (**c**) SBA-15-NCO, (**d**) SBA-15-SH, (**e**) SBA-15-Ph and (**f**) SBA-15-CH_3_.

SBA-15 and grafted functional groups: 

Figure 1a shows FT-IR spectra of pure SBA-15 after calcination and SBA-15 with loaded naproxen (acid/sodium salt). The successful synthesis and calcination of SBA-15 confirm characteristic silica vibrations at 1111, 806 and 475 cm^−1^, corresponding to Si-O-Si asymmetric (ν_as_(SiOSi)) stretching vibrations, symmetric (ν_s_(SiOSi)) stretching vibrations and bending vibration (δ(SiOSi)), respectively. In the FT-IR spectrum, there are also two broad absorption bands at 3482 and 1641 cm^−1^, which belong to the stretching and bending vibration of the OH group originating from physisorbed water molecules located in the pores and hydroxyl groups on the surface of pure SBA-15. Moreover, mentioned characteristic vibrations are present in all IR spectra of post-synthetically modified materials.

Characteristic absorption bands in the FT-IR spectra of surface-modified materials proved the presence of grafted functional groups (NH_2_, NCO, SH, Ph and CH_3_) on the surface of mesoporous silica. In all FT-IR spectra, alkoxysilanes exhibit valence symmetric and asymmetric valence CH vibrations located in the interval 2900–3000 cm^−1^ (see Figure 1). In the FT-IR spectrum of SBA-15-NH_2_ (see Figure 1b), the characteristic two N–H stretching bands of the primary amine group typically located in the 3300–3500 cm^−1^ wavenumber range are not present, as they overlap with the *ν*(OH) absorption band (3437 cm^−1^) of physisorbed water molecules. However, the presence of amine groups in the sample is confirmed by the *δ*(NH_2_) vibration at 1612 cm^−1^. The FT-IR spectrum of SBA-15 with isocyanate functional groups (SBA-15-NCO) is depicted in Figure 1c and the following absorption bands confirmed the presence of NCO groups: the strong absorption band at 2360 cm^−1^ of the ν(NCO) stretching vibration, the ν(C=O) stretching vibration located at 1650 cm^−1^ and ν(C-N) at 1560 cm^−1^. The FT-IR spectrum for material SBA-15-SH (see Figure 1d) contains a weak ν_s_(C-S) stretching vibration band at 673 cm^−1^, which confirms the presence of thiol groups on the support’s surface. The FT-IR spectrum of SBA-15-Ph (see Figure 1e) shows the presence of the phenyl group through the absorption band at 1515 cm^−1^, which corresponds to ν(C=C) stretching vibration and absorption band located at 3010 cm^−1^ of ν(C-H) of the aromatic ring. FT-IR spectrum of SBA-15-CH_3_ (see Figure 1f) shows the presence of methyl groups by ν(CH_3_) vibrations at 2960 and 2860 cm^−1^.

Drug-loaded materials: The presence of naproxen acid in pores of unmodified/modified SBA-15 is evident from absorption band at 1603 cm^−1^, which corresponds to the stretching vibration of the aromatic ring (ν(C=C)), and ν(C=O) stretching vibration around 1740 cm^−1^ in FT-IR spectra of all prepared SBA-15-X-HNAP materials (see Figure 1). IR spectra of SBA-15-X-NaNAP samples contain two characteristic asymmetric and symmetric stretching vibrations of carboxylate group about 1640 cm^−1^ for ν_as_(COO^−^) and around 1380 cm^−1^ for ν_s_(COO^−^). For comparison, the infrared spectra of pure drugs are illustrated in Figure S3 in ESI.

### 3.3. Nitrogen Adsorption/Desorption Measurements

**Figure 2:**
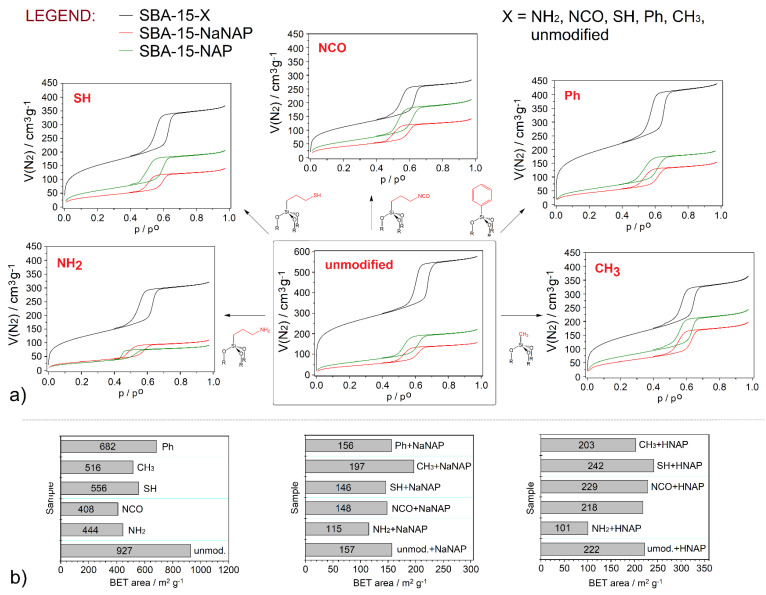
(**a**) Nitrogen adsorption/desorption isotherms measured at −196 °C of prepared materials with (**b**) the corresponding calculated BET areas.

The textural properties (surface area (*S_BET_*), pore volume (*V_p_*) and pore diameter (*d*)) of the prepared materials were determined by nitrogen adsorption/desorption measurements at −196 °C. The obtained isotherms are shown in Figure 2a, and calculated textural parameters are depicted in Figure 2b and summarized in Table 1. All measured isotherms could be classified as type *IV* by IUPAC [35] with H1 hysteresis loop in the relative pressure range of *p/p*_0_ = 0.4–0.7. As can be seen from the above values, the surface area of unmodified SBA-15 was 927 m^2^ g^−1^, and the value decreased after the grafting process. *S_BET_* area of modified materials ranged from 408 m^2^ g^−1^ (SBA-15-NCO) to 682 m^2^ g^−1^ (SBA-15-Ph). Mentioned decrease in the surface area confirms the presence of functional groups in the prepared materials, and the decline depends on the number and bulkiness of the organic group. In the case of drug-loaded samples, there was a significant decrease in *S_BET_*. Materials with stored NaNAP showed *S_BET_* surfaces in the range of 115 m^2^ g^−1^ (SBA-15-NH_2_ + NaNAP)—197 m^2^ g^−1^ (SBA-15-CH_3_ + NaNAP), and samples with encapsulated HNAP have *S_BET_* area values between 101 m^2^ g^−1^ (SBA-15-NH_2_ + HNAP)—242 m^2^ g^−1^ (SBA-15-CH_3_ + HNAP). This fact confirms the presence of drug molecules in the pores of carriers. The evolution of *S_BET_* areas for post-synthetically modified and drug-loaded materials can be arranged in the following three orders:

Surface-modified samples: SBA-15-NCO (408 m^2^ g^−1^) < SBA-15-NH_2_ (444 m^2^ g^−1^) < SBA-15-CH_3_ (516 m^2^ g^−1^) < SBA-15-SH (556 m^2^ g^−1^) < SBA-15-Ph (682 m^2^ g^−1^).

NaNAP-loaded samples: SBA-15-NH_2_ + NaNAP (115 m^2^ g^−1^) < SBA-15-SH + NaNAP (146 m^2^ g^−1^) < SBA-15-NCO + NaNAP (148 m^2^ g^−1^) < SBA-15-Ph + NaNAP (156 m^2^ g^−1^) < SBA-15-CH_3_ + NaNAP (197 m^2^ g^−1^).

HNAP-loaded samples: SBA-15-NH_2_ + HNAP (101 m^2^ g^−1^) < SBA-15-Ph + HNAP (203 m^2^ g^−1^) < SBA-15-NCO + HNAP (218 m^2^ g^−1^) < SBA-15-SH + HNAP (229 m^2^ g^−1^) < SBA-15-CH_3_ + HNAP (242 m^2^ g^−1^).

As the decreases in textural properties are more pronounced in NaNAP samples than in HNAP materials, a more considerable amount of naproxen sodium can be expected. Moreover, a comparison of *S_BET_* values indicates that an amine-modified surface, as a representative of the polar groups, exhibits a more significant amount of stored drug regardless of naproxen form (acid/sodium salt). On the other hand, the methyl group as a nonpolar functional group causes surface hydrophobicity and repulses NAP molecules leading to a smaller amount of storage drug and a larger surface area of SBA-15-CH_3_ + NaNAP/HNAP samples. It should also be noted that there was a decrease in the pore volume and pore size of the materials (see Table 1).

### 3.4. Thermogravimetric Analysis

**Figure 3:**
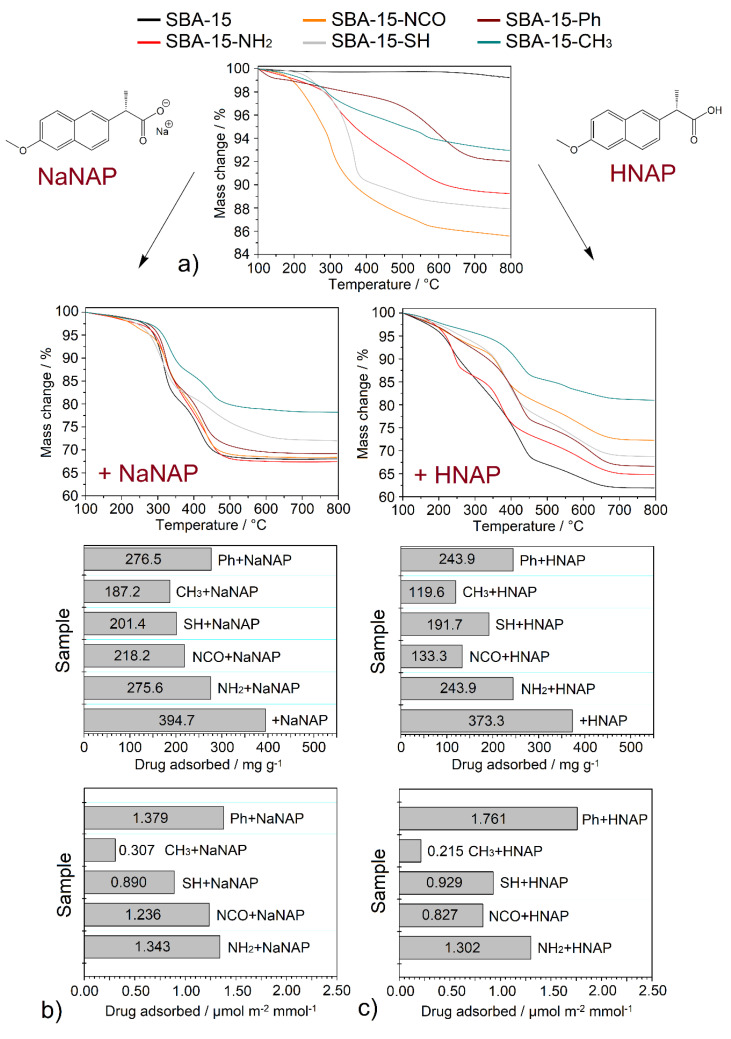
Thermogravimetric curves of (**a**) surface-modified materials and drug-loaded samples with (**b**) naproxen sodium and (**c**) naproxen acid.

Thermogravimetric (TG) analysis was used to determine the thermal stability of the prepared materials and to calculate the number of functional groups and the stored drug. All TG curves were normalized to 100 °C to eliminate the contribution of solvents, and the corresponding weight loss was adequately distributed between the mass change of the organic part and the residual mass. The measured TG curves of the prepared materials are shown in Figure 3a–c, and the calculated amount of encapsulated drug in the carriers is summarized in Table 2 and Figure 3b,c.

SBA-15 and surface-modified materials: The TG curve of SBA-15 (see Figure 3a) shows a constant course without weight change up to 640 °C. Above this temperature to 800 °C, a weight loss of 0.77 wt.% is observed, which corresponds to the SBA-15 surface dehydroxylation. For surface-modified materials, more significant weight losses were observed, which confirmed the presence of functional groups in the prepared samples (see column Grafted groups in Table 2, Figure 3a). Although the same amount of trialkoxysilane (25 mmol per gram of SBA-15) was used in the post-synthetic modification process, different numbers of functional groups were anchored on the SBA-15 surface, depending mainly on the bulkiness of the functional group. Methyl groups were the most attached, and the number of functional groups decreased to the phenyl group, as a representative of the bulkiest group. The results obtained can be arranged in the following order:

SBA-15-Ph (1.17 mmol g^−1^) < SBA-15-SH (1.61 mmol g^−1^) < SBA-15-NCO (1.72 mmol g^−1^) < SBA-15-NH_2_ (1.83 mmol g^−1^) < SBA-15-CH_3_ (4.68 mmol g^−1^).

Drug-loaded materials: In general, the amount of stored drug was calculated as the difference in weight loss of the samples containing drug and surface-modified materials (without drug). In addition, for samples containing NaNAP, corrections were made for weight loss and residual mass because the decomposition product of pure NaNAP is Na_2_CO_3_ (see Figure S4 in ESI). The obtained TG curves of NaNAP stored materials are shown in Figure 3b, and the calculated drug amounts in different units are listed in Table 2. As shown in this table, the amount of drug stored was mainly influenced by the surface area and pore volume of the materials after modification and the polarity of functional group. The most significant amount of drug was stored in unmodified SBA-15 (394.7 mg g^−1^, 1.565 mmol g^−1^), and values decreased to the methyl functional group (187.2 mg g^−1^, 0.742 mmol g^−1^). In order to better interpret the results obtained on the effect of polarity and the number of functional groups, a recalculation was performed, taking into account the mentioned parameters (see Table 2). Initially, the amounts of NaNAP in mg g^−1^ adsorbed were converted to mmol g^−1^. Subsequently, the *S_BET_* area of the modified support was taken into account according to the results summarized in Table 1. The value of stored NaNAP in mmol g^−1^ was divided by the surface area of the respective material in m^2^ g^−1^. The described calculation considers the different surface areas of the post-synthetically modified samples (in mmol m^−2^). Subsequently, further recalculation was performed to take into account the number of functional groups bound to the surface of a support. In this calculation, the amount of NaNAP in mmol m^−2^ was divided by the number of functional groups on the support surface in mmol g^−1^. The value of NaNAP molecules interacted with polar/non-polar functional groups in the matrix (in μmol m^−2^ mmol^−1^) can be obtained by the given recalculation procedure, and the results can be arranged in the following order:

SBA-15-CH_3_ + NaNAP (0.307 μmol m^−2^ mmol^−1^) < SBA-15-SH + NaNAP (0.890 μmol m^−2^ mmol^−1^) < SBA-15-NCO + NaNAP (1.236 μmol m^−2^ mmol^−1^) < SBA-15-NH_2_ + NaNAP (1.343 μmol m^−2^ mmol^−1^) < SBA-15-Ph + NaNAP (1.379 μmol m^−2^ mmol^−1^).

As can be seen from the order above, phenyl (SBA-15-Ph), animopropyl- (SBA-15-NH_2_) and isocyanatepropyl (SBA-15-NCO) modified material show the highest affinity for NaNAP molecules (between 1.2 and 1.4 μmol m^−2^ mmol^−1^). Although the -NH_2_ and -NCO groups are polar and the -Ph group is non-polar, the materials show high-affinity values. This surprising fact can be explained by the formation of hydrogen bonds of NaNAP with -NH_2_ and -NCO for SBA-15-NH_2_ and SBA-15-NCO samples and the π-π stacking interaction between -Ph and the naphthalene moiety of NaNAP. Similar results were observed in a study of the interactions of the anticancer drug Pemetrexed with phenyl- and amine-modified mesoporous silica [36]. The -SH group, which cannot form hydrogen bonds but forms dipole–dipole interactions with NaNAP, shows lower affinity. The lowest affinity was observed for SBA-15-CH_3_ material, whose hydrophobic surface repulses polar NaNAP molecules. A similar trend was observed for naproxen acid (see Figure 3c and Table 2). The solubility of the acidic naproxen form is lower than its sodium salt, 55 mg cm^−3^ (in chloroform @ 20 °C) and 76 mg cm^−3^ (in water @ 20 °C) for HNAP and NaNAP [37], respectively. This decrease in solubility was reflected in the lower amount of drug stored. Again, most drugs were encapsulated in unmodified SBA-15 (373.3 mg g^−1^, 1.621 mmol g^−1^), and values decreased to the methyl functional group (119.6 mg g^−1^, 0.519 mmol g^−1^). The trend of the surface size effect and functional groups number and thus the affinity of HNAP to surface groups was almost identical to NaNAP. The exception is the changed order of materials SBA-15-NCO and SBA-15-SH.

SBA-15-CH_3_ + HNAP (0.215 μmol m^−2^ mmol^−1^) < SBA-15-NCO + HNAP (0.827 μmol m^−2^ mmol^−1^) < SBA-15-SH + HNAP (0.929 μmol m^−2^ mmol^−1^) < SBA-15-NH_2_ + HNAP (1.302 μmol m^−2^ mmol^−1^) < SBA-15-Ph + HNAP (1.761 μmol m^−2^ mmol^−1^).

### 3.5. Solid-State NMR Spectroscopy

Solid-state NMR spectroscopy is recognized as a powerful method for providing detailed structural information on intra- and intermolecular interactions, molecular micro-segregation and on the segmental dynamics of multicomponent pharmaceutical solids. This method [38,39,40,41] is particularly useful for the systems where the active compound forms nanosized amorphous or semicrystalline domains. Therefore, primary information regarding the polymorphism of the API and the structure, composition and segmental mobility of the prepared HNAP and NaNAP systems were obtained from ^1^H MAS, *T*_1_-filtered ^13^C MAS and ^13^C CP/MAS NMR spectra. The *T*_1_-filtered ^13^C MAS NMR (^13^C MAS NMR) experiment is based on the selective detection of the ^13^C magnetization of rapidly relaxing, usually highly mobile, components. On the other hand, the ^13^C magnetization of rigid segments usually exhibits long relaxation times and thus requires long repetition delays to be effectively recovered into the thermal equilibrium. This way, signals from carbon species with a long relaxation time will be greatly diminished in the resulting *T*_1_-filtered ^13^C MAS spectra. In contrast, the ^13^C magnetization of rigid segments is selectively excited and enhanced in the ^13^C CP/MAS NMR spectra measured with short cross-polarization contact times. These techniques were used to investigate the structure of NaNAPs, and HNAPs incorporated into SBA-15 silica particles, whose efficient surface functionalization has been documented in our previous work [36].

**Figure 4:**
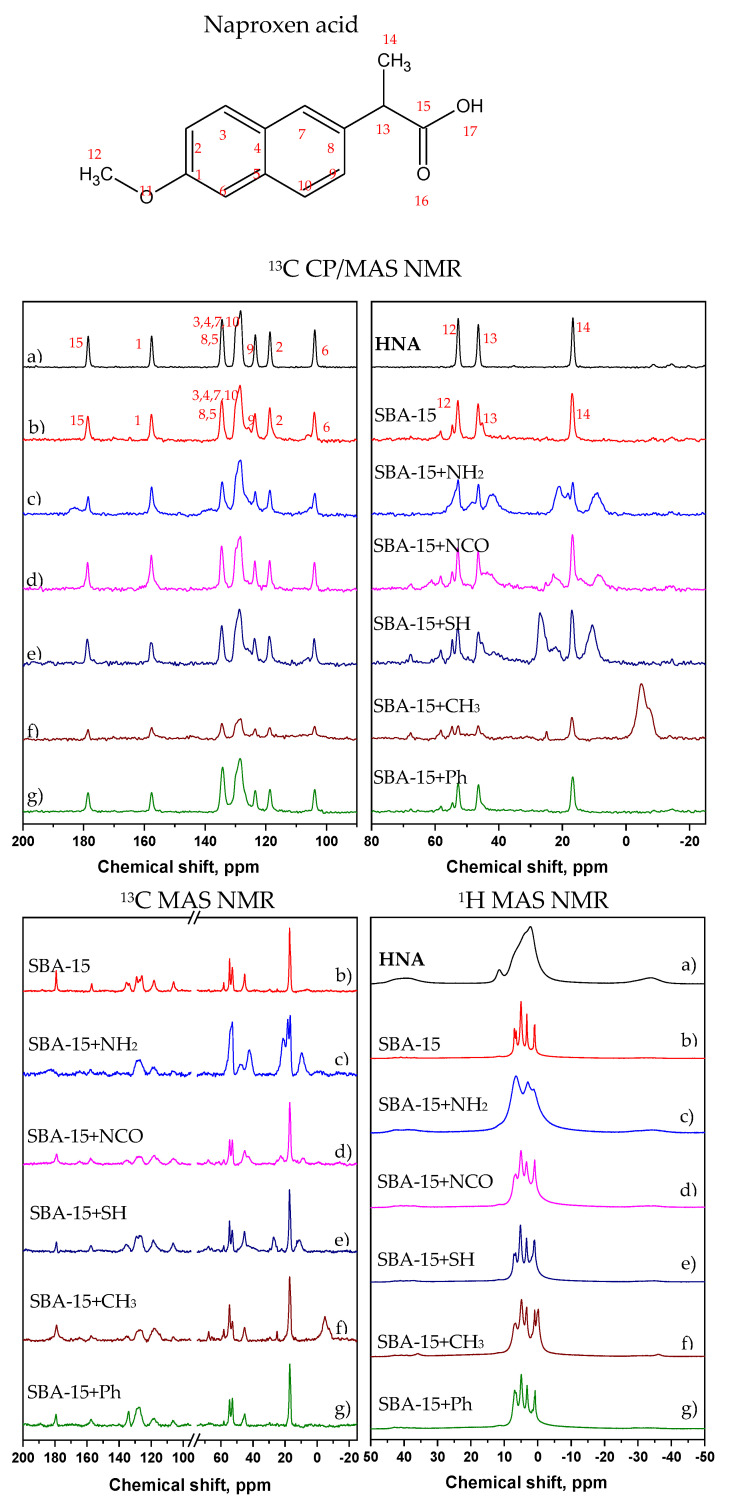
^13^C MAS and CP/MAS NMR, ^1^H MAS NMR spectra. Detailed regions from 200 to 90 ppm and from 80 to -25 ppm of ^13^C CP/MAS NMR spectra HNAP systems. The neat (a) HNAP; (b) SBA-15 + HNAP; (c) SBA-15-NH_2_ + HNAP; (d) SBA-15-NCO + HNAP; (e) SBA-15-SH + HNAP; (f) SBA-15-CH_3_ + HNAP; and (g) SBA-15-Ph + HNAP.

**Figure 5:**
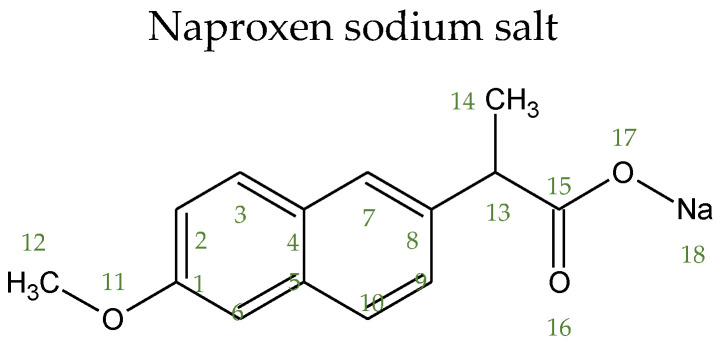

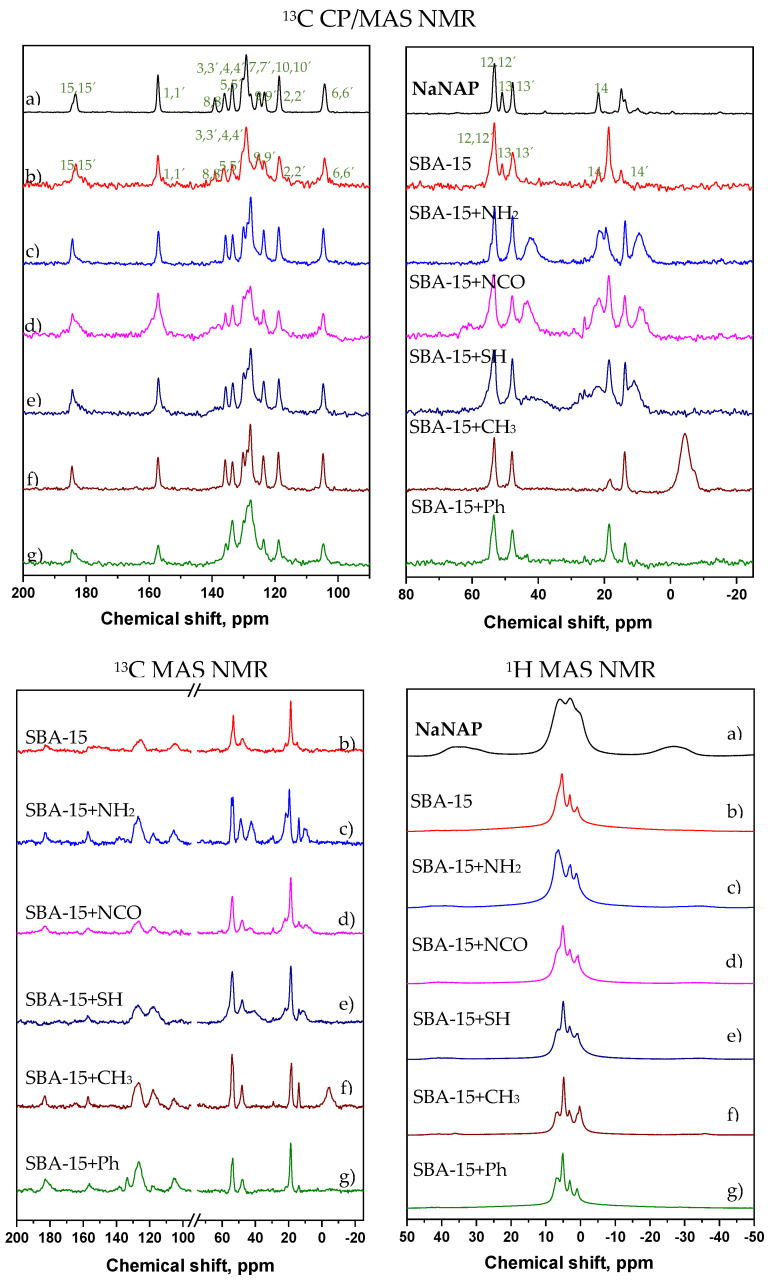
^13^C MAS and CP/MAS NMR, ^1^H MAS NMR spectra. Detailed regions, from 200 to 90 ppm and from 80 to -25 ppm of ^13^C CP/MAS NMR spectra NaNAPs systems. The neat (a) NaNAP; (b) SBA-15 + NaNAP; (c) SBA-15-NH_2_ + NaNAP; (d) SBA-15-NCO + NaNAP; (e) SBA-15-SH + NaNAP; (f) SBA-15-CH_3_ + NaNAP; and (g) SBA-15-Ph + NaNAP.

Specifically, the ^13^C CP/MAS NMR spectra of neat HNAP and NaNAP compounds (Figure 4 and Figure 5, respectively) are characterized by narrow signals, which indicates their highly ordered crystalline state [42]. The ^13^C CP/MAS NMR spectra of HNAP systems (Figure 4, upper boxes) show that a considerably high fraction of HNAP reflected by the narrow ^13^C CP/MAS NMR signals remains almost unchanged in the crystalline state. However, the observed slight broadening of these signals and, in some cases, even the presence of a new set of low-intensity broad signals indicates the formation of a small amount of amorphous structurally disordered fractions of HNAP. These fractions, as strongly immobilized, form relatively large domains that are probably located mainly on the surface of SBA-15 particles [43]. The lower intensity of HNAP signals for the SBA-15-CH_3_ + HNAP system indicates a lower amount of crystalline (rigid) HNAP compared to the other systems in this series.

However, the single-pulse ^13^C and ^1^H MAS NMR spectra (Figure 4, bottom panels left and right, respectively) further revealed a considerable presence of an additional phase of HNAP. Except for the system SBA-15-NH_2_ + HNAP, this fraction is always reflected by the narrow signals, which indicates its high molecular mobility. This is particularly apparent in ^1^H MAS NMR spectra. In agreement with the previously published findings [43], these narrow signals can be attributed to HNAP molecules that are predominantly located within the pores of the SBA-15 carrier. An exception is the system SBA-15-NH_2_ + HNAP, which is in both types of single-pulse spectra (^13^C and ^1^H MAS NMR) reflected by the relatively broad signals. This broadening of the signals then reflects an immobilization of HNAP molecules. These molecules instead form large amorphous domains or are tightly incorporated on the surface of pores of the SBA-15 carrier.

Similarly, as in the previous case, in SBA-15 + NaNAP systems, the recorded solid-state NMR spectra (Figure 5) revealed the presence of various fractions of the active compound. While the narrow signals detected in ^13^C CP/MAS NMR spectra (Figure 5, upper panels) indicate the presence of a residual fraction of crystalline naproxen sodium salt, the broad ones resonating at similar resonance frequencies reflect the coexistence of NaNAP molecules in strongly disordered and amorphous domains. Such a transformation is clearly apparent for the SBA-15-NCO + NaNAP system.

Whereas the residual crystalline fraction is predominantly located on the surface of the SBA-15 carrier, the amorphous domains can at least partly also occupy the largest pores. In addition to these relatively rigid fractions, the narrow signals recorded in single-pulse ^1^H and ^13^C MAS NMR spectra indicate the presence of highly mobile molecules of the active compound that are located inside the pores, where they lost the majority of intermolecular interactions. Compared with HNAP, the tendency toward the API transformation and amorphization is notably higher for the sodium salt form.

**Figure 6:**
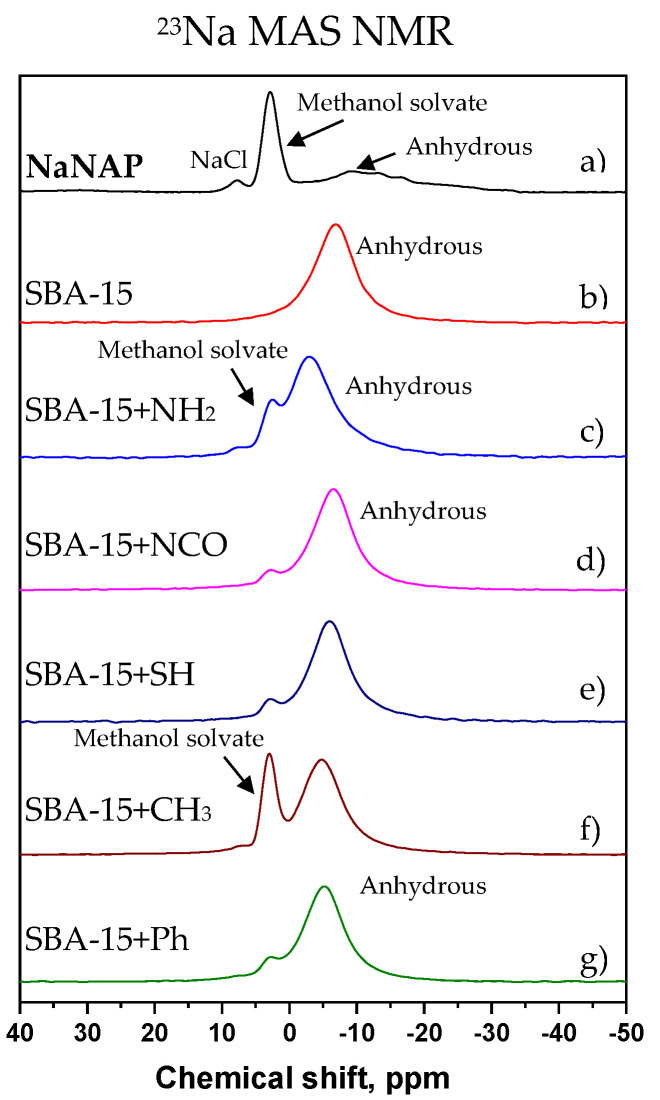
^23^Na MAS of NaNAPs systems. The neat (a) NaNAP; (b) SBA-15 + NaNAP; (c) SBA-15-NH_2_ + NaNAP; (d) SBA-15-NCO + NaNAP; (e) SBA-15-SH + NaNAP; (f) SBA-15-CH_3_ + NaNAP; and (g) SBA-15-Ph + NaNAP.

Extensive structural transformation of naproxen sodium salt is further demonstrated in ^23^Na MAS NMR spectra (see Figure 6).

Whereas the original crystalline NaNAP system predominantly exists in the form of methanol solvate and the anhydrous form, when incorporated into the surface-modified SBA-15 carriers, the original narrow signal at ca. 3 ppm reflecting crystalline NaNAP [44] methanol solvate mostly disappears, and a new broad and featureless signal at −5 ppm becomes more apparent. According to the literature data, the broad signal at −5 ppm can be attributed to the amorphous anhydrous form of NaNAP [45]. It is interesting how surface modification affects the tendency to API transformation. Whereas no residual methanol solvate fraction is present in the neat SBA-15 carrier, a relatively large amount of NaNAP in the crystalline methanol solvate form is found for the SBA-15-CH_3_ system. This finding derived from ^23^Na MAS NMR data is relatively consistent with ^13^C CP/MAS NMR spectrum.

### 3.6. Drug Release Properties

**Figure 7:**
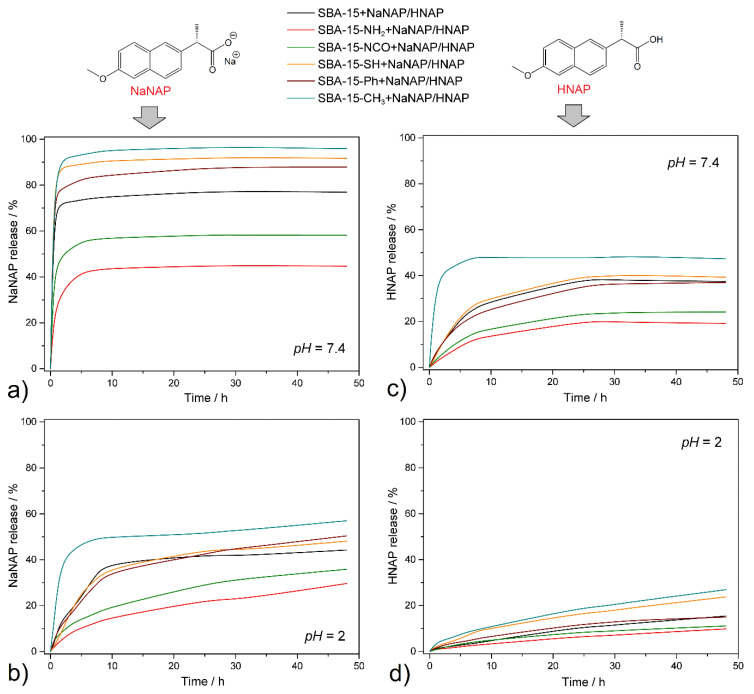
Time-depended drug release curves at *t* = 37 °C for (**a**) NaNAP at *pH* = 7.4, (**b**) NaNAP at *pH* = 2, (**c**) HNAP at *pH* = 7.4 and (**b**) HNAP at *pH* = 2.

In vitro drug release experiments of naproxen (acid and salt forms) were realized under different *pH* conditions. In all experiments, physiological solutions with different *pH* were used: *pH* = 2 was chosen as the environment of gastric fluid and *pH* = 7.4 as the simulated intravenous fluid/small intestine medium. The temperature was set at *t* = 37 °C as the physiologic temperature of the human body. The total amount of released naproxen was determined in selected time intervals—0.5, 1.5, 3.5, 5.5, 7.5, 9.5, 24, 28, 32, and 48 h—and analyzed by UV spectroscopy. The drug released amount was determined using calibration curves measured and calculated at selected *pH* (*λ_max_* = 231 nm, see calibration curves in Figure S1 in ESI). For the percentual values of the released amount of naproxen, the calculated data from the thermogravimetric analysis were used (see Table 2 in Section 3.4 Thermogravimetric analysis). The discussion of the results obtained is divided into four parts depending on the drug form and the *pH* of the environment, as shown in Figure 7.

**Figure 8:**
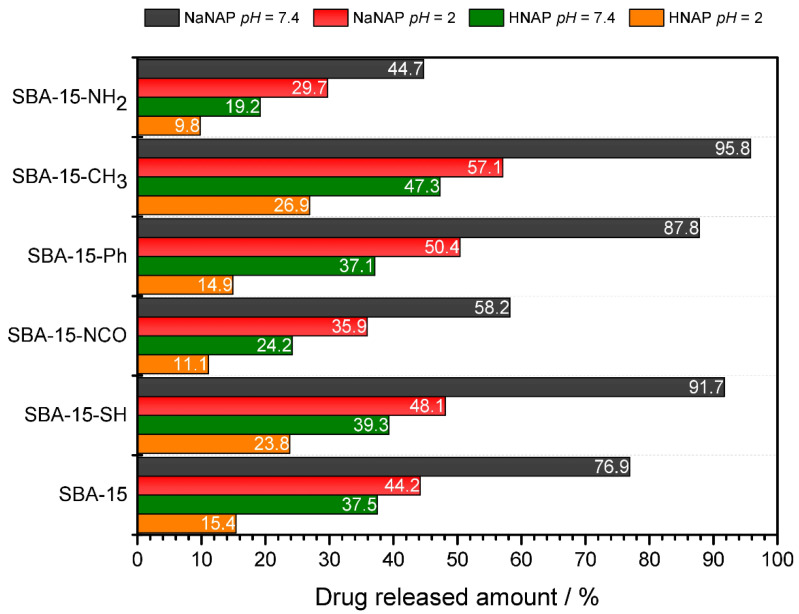
Maximum drug released amounts from the prepared materials after 48 h at *t* = 37 °C and different *pH*.

**1. NaNAP, *pH* = 7.4:** Figure 7a illustrates the drug release curve of NaNAP at *pH* = 7.4 and *t* = 37 °C. It is evident from Figure 6a that the drug release occurs rapidly and abruptly during the first hours of release and the total release occurs after 10 h of experiments. Rapid release occurs from the materials SBA-15-CH_3_ + NaNAP, SBA-15-SH + NaNAP and SBA-15 + NaNAP, which is evident from the course and shape of the curve. On the other hand, from SBA-15-NH_2_ + NaNAP, SBA-15-NCO + NaNAP and SBA-15-Ph + NaNAP, the release was slightly inhibited due to intermolecular interactions between drug molecules and surface functional groups on SBA-15. The amounts of total drug released after 48 h are shown in Figure 8, and the values obtained can be arranged in the following order:

SBA-15-CH_3_ + NaNAP (95.8%) > SBA-15-SH + NaNAP (91.7%) > SBA-15-Ph + NaNAP (87.8%) > SBA-15 + NaNAP (76.9%) > SBA-15-NCO + NaNAP (58.2%) > SBA-15-NH_2_ + NaNAP (44.7%).

The -NH_2_ and -NCO groups can form hydrogen bonds with NaNAP, and the -Ph functional group forms *π-π* interactions with the naphthalene moiety of the drug, as confirmed in previous studies using aromatic drugs such as pemetrexed [36], ibuprofen [46], doxorubicin [47] and cumminaldehyde [48]. It is clear from the results that the methyl (-CH_3_) and thiol (-SH) groups do not interact with the drug. In addition, the -CH_3_ group significantly increases the hydrophobicity of the surface, which significantly repulse the hydrophilic drug (NaNAP), which is reflected in its rapid release rate and the highest NaNAP amount released among all samples. As can be seen from the results obtained, under the given experimental conditions (*pH* = 7.4, *t* = 37 °C), a large amount of drug was released, which was controlled not only by the type of functional groups but also by the solubility of NaNAP, which is 99 mg NaNAP / 1 cm^3^ at *t* = 37 °C and *pH* = 6.8 in water [49].

**2. NaNAP, *pH* = 2:** As can be seen from Figure 7b, at *pH* = 2 and *t* = 37 °C, approximately half of the NaNAP was released from all materials compared to *pH* = 7.4. In addition to the smaller drug release, a change in the shape of the release curve can also be observed. The material SBA-15-CH_3_ + NaNAP shows a burst release of NaNAP in the first 8 h of the experiment, while the shape of the isotherms for the remaining materials shows a gradual release within 28 h. No further drug release was observed at subsequent time intervals, and solution saturation occurred. Based on the results obtained, the amount of NaNAP released can be classified into the following order, depending on the materials:

SBA-15-CH_3_ + NaNAP (57.1%) > SBA-15-Ph + NaNAP (50.4%) > SBA-15-SH + NaNAP (48.1%) > SBA-15 + NaNAP (44.2%) > SBA-15-NCO + NaNAP (35.9%) > SBA-15-NH_2_ + NaNAP (29.7%).

As can be seen from this series, a similar trend was observed at *pH* = 2 compared to *pH* = 7.4 (see Figure 7a,b). The functional groups interacted with the drug, manifested by a gradual drug release (curve shape) and the amount of NaNAP released. The *pH* of the environment had a significant effect on the total amount of drug released. At *pH* = 2, protonation of naproxen sodium salt and its transformation to acid was observed, which led to reduced solubility associated with smaller drug releases. This statement is evidence of the protonation constant *pK_a_* = 4.19 for naproxen molecules [28]. The solubility of sodium naproxen is 14 mg NaNAP / 1 cm^3^ at *t* = 37 °C and *pH* = 1.3 in water [49].

**3. HNAP, *pH* = 7.4:** It is well known that NSAIDs (acid form) have poor solubility in water compared to their salt forms, which was also observed in our experiment. In addition to the influence of functional group type, the achieved results were also controlled by drug solubility. As shown in Figure 7c, the course of the curves and the total amount of drug released are similar to those of NaNAP at *pH* = 2 experiments within 28 h. The maximum amounts of HNAP released after 48 h can be arranged in the following order:

SBA-15-CH_3_ + HNAP (47.3%) > SBA-15-SH + HNAP (39.3%) > SBA-15 + HNAP (37.5%) > SBA-15-Ph + HNAP (37.1%) > SBA-15-NCO + HNAP (24.2%) > SBA-15-NH_2_ + HNAP (19.2%).

Again, the same trend of maximum drug release can be observed depending on the type of functional groups. The solubility of HNAP is lower compared to NaNAP, concretely 3.4 mg HNAP / 1 cm^3^ at *pH* = 7.4 and *t* = 35 °C in water [50,51]; this was reflected in smaller amounts of drug released.

**4. HNAP, *pH* = 2:** The lowest drug released amounts were experimentally determined for HNAP and *pH* = 2, *t* = 37 °C (see Figure 7d and Figure 8). As can be seen from the drug release isotherms of the prepared materials, after 48 h there was no saturation of the solution, and thus no total release of HNAP from the matrixes and all drug release isotherms had a rising trend (see Figure 7d). The maximum amount released was from the material SBA-15-CH_3_ + HNAP, which gradually decreased to SBA-15-NH_2_ + HNAP, and the values can be arranged in the following order:

SBA-15-CH_3_ + HNAP (26.9%) > SBA-15-SH + HNAP (23.8%) > SBA-15 + HNAP (15.4%) > SBA-15-Ph + HNAP (14.9%) > SBA-15-NCO + HNAP (11.1%) > SBA-15-NH_2_ + HNAP (9.8%).

The influence of intermolecular interactions was also observed under the given experimental conditions (*pH* = 2, *t* = 37 °C): formation of hydrogen bonds (SBA-15-NH_2_ + HNAP and SBA-15-NCO + HNAP) and *π-π* interactions (SBA-15-Ph + HNAP), but also the solubility of the drug itself had a significant effect. The solubility of naproxen acid is 0.03 mg HNAP/1 cm^3^ at *pH* = 2 and *t* = 35 °C in water [50,51].

Figure 8 shows the maximal amounts of released naproxen after 48 h at *t* = 37 °C based on environment *pH*. As can be seen, the best conditions for the controlled release of naproxen, due to the drug activity in a human body treatment such as a strong painkiller, are when it is encapsulated as a naproxen sodium salt in SBA-15-CH_3_ or SBA-15-SH for rapid release in an intravenous environment and the small intestine. In contrast, SBA-15-NH_2_, SBA-15-NCO and SBA-15-Ph showed slower and lower drug release rates and amounts. In this case, the human stomach is protected by slow drug release from the matrix, which is a prevention measure against stomach ulcers caused by NSAID types. The best choice for inflammatory diseases is a very-slow-release system, where naproxen acid is used.

**Figure 9:**
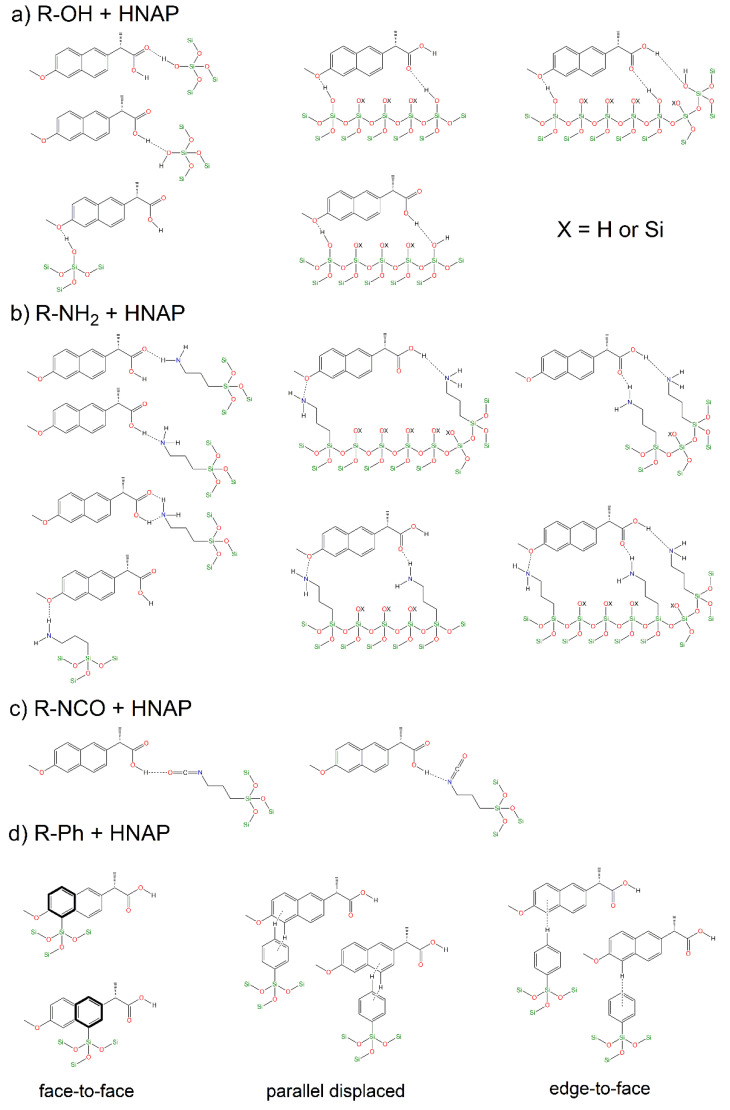
Possible formation of hydrogen bonds between naproxen acid and (**a**) hydroxyl, (**b**) propylamine, (**c**) propylisocyanate groups, and (**d**) π-π interaction of HNAP with a phenyl group.

As described above, several factors influenced the final course and maximum amounts of drugs released from the prepared DDSs:

(a) **Surface polarity**—For the silica material, the polarity of its surface and the drug becomes an essential factor when considering its versatility in drug release and delivery. In general, it can be concluded that polar groups (-NH_2_, -NCO, -SH and -OH in pure SBA-15) significantly influenced the total released amounts of HNAP and NaNAP. In most experiments, amine, isocyanate and hydroxyl groups had the most significant influence, affecting the surface’s polarity to varying degrees. Since the well-known rule “like dissolves” applies, the aforementioned groups interacted more with naproxen sodium, which is more polar compared to the drug in its acid form. From the point of view of the non-polar groups used, phenyl and methyl, the -Ph group has a higher polarity compared to CH_3_, which was also reflected in the experimental results of drug release. Depending on the functional group used, the polarity of the surface can be divided into the following series: -OH > -NH_2_ > -NCO > -SH > -Ph > -CH_3_. The experimental results show that the rate and amount of drug released depended on the polarity of the matrices: more hydrophilic matrices could store larger amounts of NaNAP and HNAP and promote slower drug release. On the contrary, hydrophobic groups, specifically the methyl group, decreased the surface’s polarity, resulting in a burst release of the selected drugs. There is also another important factor related to the polarity that had an impact on the course of the release experiments, and that was intermolecular interactions.
(b) **Intermolecular interactions**—The polarity of the carrier surface is also related to the intermolecular interactions between functional groups of grafted molecules and drug molecules (NaNAP and HNAP). Of the polar groups, hydroxyl, amine, and isocyanate can form hydrogen bonds with HNAP (see Figure 9). Based on the formation of hydrogen bonds, it is possible to explain the higher stored amounts of drugs and increasing drug release in the simulated model solutions. As can be seen from Figure 9a,b, the amine and hydroxyl groups have more opportunities to form hydrogen bonds with HNAP than isocyanate. Although the isocyanate group contains two donor atoms (N and O), due to the geometry of the molecule and the electronegativity of the atoms, oxygen donation is preferred. This does not mean that forming an O-H···N hydrogen bond is impossible (see Figure 9c). Although the thiol group does not form hydrogen bonds, the contribution of the dipole–dipole interaction is significant, and the experimental results from drug release can be explained through the mentioned interaction. An interesting trend was observed for non-polar groups, where the phenyl group exceeds the polar thiol group. The mentioned observation can be explained based on the formation of *π-π* interactions (face-to-face, parallel displaced, edge-to-face, see Figure 9d) [36], which inhibit the rate and total amount of released drugs compared to the methyl group, which is also a representative of non-polar groups.
(c) ***pH and drug solubility—***The *pH* of the environment also significantly affects the amount of drug released (see comparison Figure 7a,b for NaNAP; Figure 7c,d for HNAP). *pH* has the most significant effect on drug solubility and intermolecular interactions. Based on the literature review, it can be concluded that sodium salt has almost five times higher solubility in a nearly neutral environment compared to acidic *pH* (NaNAP solubility: 14 mg cm^−3^ at *pH* = 1.3 and 99 mg cm^−3^ at *pH* = 6.8 [49]). In the case of naproxen acid, a similar trend was observed, as solubility in a slightly alkaline environment is up to 113 times higher compared to acidic *pH* (HNAP solubility: 0.03 mg cm^−3^ at *pH* = 1.2 and 3.4 mg cm^−3^ at *pH* = 7.4 [50,51]). Since the value of the naproxen protonation constant is *pKa* = 4.19 [28], in an acidic environment at *pH* = 2, the sodium salt of naproxen is transformed to acid, which can be interpreted as the reduced released amounts of NaNAP from the carriers (see the comparison of maximum NaNAP released amounts, black and red columns in Figure 8 for *pH* = 7.4 and *pH* = 2, respectively). The opposite point of view can be applied to the slightly soluble form of naproxen HNAP, which although showing low solubility at acidic *pH*, in the presence of a slightly alkaline environment deprotonation of carboxyl groups occurs (*pH* = 7.4 > *pKa* = 4.19) and solubility increases (see the comparison of maximum HNAP released amounts, green and orange columns in Figure 8 for *pH* = 7.4 and *pH* = 2, respectively). However, it should be noted that *pH* also affects the formation or disruption of intermolecular interactions. For example, the propylamine group has a protonation constant value of ~4, which means that at *pH* = 2, its protonation occurs (-CH_2_-CH_2_-CH_2_-NH_2_ -> -CH_2_-CH_2_-CH_2_-NH_3_^+^). The surface of SBA-15-NH_2_ is positively charged at acidic *pH*, and in addition, the nitrogen atom does not contain a free electron pair. Therefore, amine groups can no longer act as an acceptor for hydrogen atoms in the formation of hydrogen bonds.
(d) **Drug crystallinity**—The crystallinity of the drug also affects the drug’s solubility, as the dissolution process consists of two essential processes, namely, the overcoming of the lattice energy of the compound’s crystal structure and the subsequent solvation energy that is released during the formation of the solvation shell. In previous studies, it was shown that when drugs are stored in porous materials, the drug is formed in an amorphous phase, in which it is not necessary to overcome the lattice energy, and thus the solubility of the drug increases [52,53]. In our study, the ^13^C CP/MAS NMR spectra of the SBA-15-X+HNAP/NAP materials showed that the drugs are in the carriers in crystalline and amorphous forms, which also had an impact on the results of the drug release experiments.

### 3.7. Drug Release Kinetic Study

The data obtained from drug release were used for the kinetic study (determination of kinetic model and naproxen release rate) based on different mathematical models: zero-order, first-order, Higuchi and Hixson–Crowell models. Calculated release rate constants and coefficients of determination *R^2^* are summarized in Table 3. The first-order mathematical model is usually used to describe drug delivery systems with a difficult mechanism of drug release from the matrix, but it can be applied to porous systems with water-soluble drugs. The Higuchi model can be applied to planar systems with diverse geometrics and porous systems. The difference between these two models is that Higuchi’s mathematical model can be used for matrices with drug diffusion only in one dimension. Moreover, the concentration in the matrix must be higher than drug solubility. Particles of the matrix must be smaller than the system thickness and rigid. The last condition concerns drug diffusion, which must have a constant releasing time. The Hixson–Crowell model can be used for drug release from a specific matrix, such as a granular medicament dosage. Moreover, it can be applied to water-soluble drugs such as powder dissolution. The last applied model used in the present study, the zero-order release kinetics model, is suitable for the constant drug release from specific DDSs based on osmosis and low-soluble drugs [54].

As can be seen from the calculated *R^2^* values (see Table 3), zero- and first-order models are not suitable for describing drug release from our prepared carriers. The *R^2^* values for the zero-/first-order mathematical model ranged from 0.1996 to 0.9802. Based on the obtained values, it can be concluded that drug release does not occur through these mechanisms. The Higuchi model can be applied to the release rate of naproxen acid and naproxen sodium salt from prepared DDSs. In most cases, the *R^2^* coefficient is over 0.8 for the Higuchi model. The samples displayed one-dimension diffusion, which is in good agreement with the SBA-15 structure (1D hexagonal channels), and hydrophobic particles provide the rigidity of structures.

## 4. Conclusions

In the presented study, mesoporous silica SBA-15 was prepared, and its surface was modified with various polar and non-polar groups. Polar groups are represented by propylamine (-NH_2_), propylisocyanate (-NCO), and propylthiol (-SH) and non-polar groups, by methyl (-CH_3_) and phenyl (-Ph). The modified materials were studied by combining TEM, IR, TG, nitrogen adsorption, and ssNMR experiments, confirming the presence of used ligands on the surface of samples and their occurrence. It was shown that bulky ligands affect their surface appearance significantly. Phenyl groups (1.17 mmol g^−1^) were the least attached to the surface of SBA-15, and methyl groups (4.68 mmol g^−1^) were the most represented. Subsequently, the drug naproxen, in the form of acid (NHAP) and sodium salt (NaNAP), was encapsulated in the materials. The amount of drug stored was determined from the TG based on the weight differences between the surface-modified materials and the materials with a loaded drug. Several factors were considered when calculating the amount of drugs stored, such as the number of surface groups and the surface area (*S_BET_*) of the modified materials. Regardless of the drug used (NaNAP and HNAP), the most significant amount of drug was stored in the phenyl-modified material (1.379 *µ*mol m^−2^ mmol^−1^ for NaNAP and 1.761 *µ*mol m^−2^ mmol^−1^ for HNAP) and the smallest quantity in the methyl-modified sample (0.307 *µ*mol m^−2^ mmol^−1^ for NaNAP and 0.215 *µ*mol m^−2^ mmol^−1^ for HNAP). Subsequently, in vitro drug release experiments were performed at *t* = 37 °C (average human body temperature) and two different *pH* values, *pH* = 7.4 as simulated body fluid and *pH* = 2 as simulated gastric fluid. In general, the amounts and rate of drug release were affected by the solubility of NaNAP and HNAP. As naproxen sodium is more soluble in water, more NaNAP was released than naproxen acid (HNAP). The intermolecular interactions between the functional groups on the SBA-15 surface and the drug molecules affected the total amount released and the shape of drug release isotherms. Because the -NH_2_ and -NCO functional groups can form hydrogen bonds and the -Ph group *π-π* interactions, the mentioned intermolecular interactions prolonged drug release and reduced the total amount released. In the case of the -CH_3_ group, which significantly increases the surface’s hydrophobicity, the rates and amount of drug released were highest regardless of the drug form. The third factor that significantly affected drug release experiments was *pH*. It can be stated that a higher rate and amount of drug released was observed at *pH* = 7.4 compared to *pH* = 2, which is also related to drug solubility. Subsequently, a drug release kinetic study was performed, where different kinetic models for fitting the experimentally obtained drug release curves were used: zero- and first-order, Higuchi and Hixson-Crowell. The best results were obtained for the Higuchi model that best describes the results and is in good agreement with the structure of a prepared system, a porous solid with drug diffusion in one dimension (1D hexagonal channels in SBA-15). The performed experiments and findings conclude that release rate and the amount of released naproxen depend strongly on intermolecular interactions in DDSs, hydrophilic/hydrophobic surface, *pH*, drug form, and solubility.

## Figures and Tables

**Table 1:** Calculated textural parameters (surface area (*S_BET_*), pore volume (*V_p_*) and pore diameter (*d*)) from nitrogen adsorption/desorption measurements measured at −196 °C using BJH (Barrett–Joyner–Halenda) method.

Samples	*S_BET_* [m^2^ g^−1^]	Textural Parameters *V_p_* [cm^3^ g^−1^]	*d * [nm]
SBA-15	927	0.70	6.5
SBA-15-NH_2_	444	0.45	5.6
SBA-15-NCO	408	0.39	5.6
SBA-15-SH	556	0.47	5.5
SBA-15-CH_3_	516	0.47	6.0
SBA-15-Ph	682	0.55	5.9
SBA-15 + NaNAP	157	0.26	5.0
SBA-15-NH_2_ + NaNAP	115	0.18	4.6
SBA-15-NCO + NaNAP	148	0.24	4.8
SBA-15-SH + NaNAP	146	0.23	4.8
SBA-15-CH_3_ + NaNAP	197	0.33	5.2
SBA-15-Ph + NaNAP	156	0.26	5.0
SBA-15 + HNAP	222	0.35	5.4
SBA-15-NH_2_ + HNAP	101	0.15	4.2
SBA-15-NCO + HNAP	218	0.32	5.2
SBA-15-SH + HNAP	229	0.35	5.3
SBA-15-CH_3_ + HNAP	242	0.36	5.5
SBA-15-Ph + HNAP	203	0.32	5.1

**Table 2:** Calculated amounts of loaded NaNAP/HNAP in different units (mass fraction, mg of drug per gram of DDS, mmol of drug per gram of DDS, µmol of drug per one square meter of DDS’s surface and µmol of drug per one square meter of DDS surface per mmol of surface-grafted functional group).

Samples after Drug Loading	Organic Part [wt.%]	Grafted Groups [wt.%] [mmol g^−1^]		[wt.%]	[mg g^−1^]	Drug [mmol g^−1^]	[μmol m^−2^]	[μmol m^−2^ mmol^−1^]
SBA-15 + NaNAP	31.95	0.77	-	31.18	394.7	1.565	1.688	-
SBA-15-NH_2_ + NaNAP	32.54	10.77	1.83	21.77	275.6	1.093	2.461	1.343
SBA-15-NCO + NaNAP	31.66	14.42	1.72	17.24	218.2	0.865	2.120	1.236
SBA-15-SH + NaNAP	28.03	12.12	1.61	15.91	201.4	0.798	1.435	0.890
SBA-15-CH_3_ + NaNAP	21.82	7.03	4.68	14.79	187.2	0.742	1.438	0.307
SBA-15-Ph + NaNAP	30.82	8.98	1.17	21.84	276.5	1.096	1.607	1.379
SBA-15 + HNAP	38.10	0.77	-	37.33	373.3	1.621	1.749	-
SBA-15-NH_2_ + HNAP	35.16	10.77	1.832	24.39	243.9	1.059	2.385	1.302
SBA-15-NCO + HNAP	27.75	14.42	1.715	13.33	133.3	0.579	1.419	0.827
SBA-15-SH + HNAP	31.29	12.12	1.612	19.17	191.7	0.833	1.498	0.929
SBA-15-CH_3_ + HNAP	18.99	7.03	4.677	11.96	119.6	0.519	1.006	0.215
SBA-15-Ph + HNAP	33.37	8.98	1.165	24.39	243.9	1.059	2.052	1.761

**Table 3:** Calculated kinetic model fitting parameters for NaNAP and HNAP release data at different *pH*.

			Zero-Order	First-Order	Higuchi	Hixson–Crowell
Sample	Drug	*pH*	*k_0_* (mol.dm^−3^.h^−1^)	*k_1_* (h^−1^)	*k_h_* (h^−0.5^)	*k_HC_* (h^−1/3^)
			r^2^	r^2^	r^2^	r^2^
**SBA-15**	HNAP	2	0.2279 0.9317	0.0513 0.6366	1.3571 0.9033	0.0264 0.7705
		7.4	0.2711 0.6892	0.0360 0.4683	2.9809 0.8142	0.0226 0.5538
	NaNAP	2	0.3025 0.7025	0.0274 0.4853	3.7152 0.72532	0.0198 0.5624
		7.4	0.1415 0.4739	0.0025 0.4563	1.6926 0.9243	0.0033 0.4622
**SBA-15-CH_3_**	HNAP	2	0.4652 0.9556	0.0492 0.7243	2.8477 0.9263	0.0332 0.8274
		7.4	0.8328 0.7644	0.0348 0.5537	8.7373 0.8611	0.0323 0.6254
	NaNAP	2	0.9764 0.7957	0.0362 0.5514	9.6945 0.9000	0.0349 0.6452
		7.4	0.9969 0.6688	0.0282 0.6499	12.585 0.9748	0.0031 0.6563
**SBA-15-SH**	HNAP	2	0.3090 0.9303	0.0481 0.7121	1.9711 0.9335	0.0286 0.8145
		7.4	0.3892 0.9023	0.0372 0.6934	2.3405 0.8943	0.0350 0.9123
	NaNAP	2	0.2045 0.5632	0.0542 0.6452	1.1402 0.7542	0.0495 0.0642
		7.4	0.2885 0.3685	0.0082 0.3198	1.3863 0.9992	0.0089 0.3360
**SBA-15-NCO**	HNAP	2	0.4419 0.9672	0.0442 0.7642	3.0243 0.9774	0.0306 0.8583
		7.4	0.8003 0.7264	0.0422 0.4910	7.0343 0.8696	0.0356 0.5878
	NaNAP	2	0.8908 0.6161	0.0342 0.3871	1.0696 0.6847	0.0321 0.4747
		7.4	0.0971 0.5177	0.0022 0.4956	1.6547 0.8343	0.0025 0.5029
**SBA-15-Ph**	HNAP	2	0.1998 0.9802	0.0522 0.8092	1.0731 0.8680	0.0261 0.8935
		7.4	0.4119 0.7523	0.0390 0.5012	3.8876 0.8914	0.0273 0.6062
	NaNAP	2	0.5382 0.9147	0.0426 0.6269	4.1371 0.9822	0.0317 0.7465
		7.4	0.1538 0.2742	0.0025 0.2558	5.8387 0.6690	0.0033 0.6168
**SBA-15-NH_2_**	HNAP	2	0.3229 0.9787	0.0565 0.8116	1.5500 0.7957	0.0321 0.8977
		7.4	0.1310 0.2336	0.0075 0.1996	7.5237 0.9598	0.0065 0.2105
	NaNAP	2	0.2422 0.4010	0.0153 0.2618	0.0125 0.3030	6.9673 0.2326
		7.4	0.1295 0.3672	0.0174 0.3506	2.6825 0.9712	0.0024 0.3561

## Data Availability

Data available in a publicly accessible repository.

## Supplementary Materials

The following supporting information can be downloaded at: https://www.mdpi.com/article/10.3390/jfb13040275/s1, Figure S1 Calibration curves of naproxen sodium and naproxen acid at different pH obtained from UV measurements used for determination of total released amount of drug. Figure S2 TEM images of prepared SBA-15 material: (a) a shape of particles and a view along (b) alo, ng and (c) parallel direction to the pores. Figure S3 IR spectra of (a) naproxen acid and (b) naproxen sodium. Figure S4 TG curves of pure drugs: naproxen sodium and naproxen acid measured in an air atmosphere.
