# Supplementary material for: Adsorption and Release Properties of Drug Delivery System Naproxen-SBA-15: Effect of Surface Polarity, Sodium/Acid Drug Form and pH
Source: J Funct Biomater. 2022 Dec 5;13(4):275. doi: 10.3390/jfb13040275 (PMC9781637; doi:10.3390/jfb13040275)
Supplement: Supplementary file 1 [file jfb-13-00275-s001.zip › jfb-2053215-supplementary.pdf]

Adsorption and Release Properties of Drug Delivery System Naproxen - SBA-15: Effect of Surface Polarity, Sodium/Acid Drug Form and *pH*

Ľuboš Zauška[a], Eva Beňová[a], Martina Urbanová[b], Jíří Brus[b], Vladimír Zeleňák[a], Virginie Hornebecq[c], Miroslav Almáši[a]

[a] Department of Inorganic Chemistry, Faculty of Science, P. J. Šafárik University, Moyzesova 11, SK-041 01 Košice, Slovak Republic

[b] Laboratory of Solid State NMR Spectroscopy, Department of Macromolecular Chemistry, Academy of Sciences of the Czech Republic, Heyrovsky square 2, CZ-162 06 Prague, Czech Republic

[c] Aix-Marseille University, CNRS, MADIREL, F-133 97 Marseille, France

Figures:

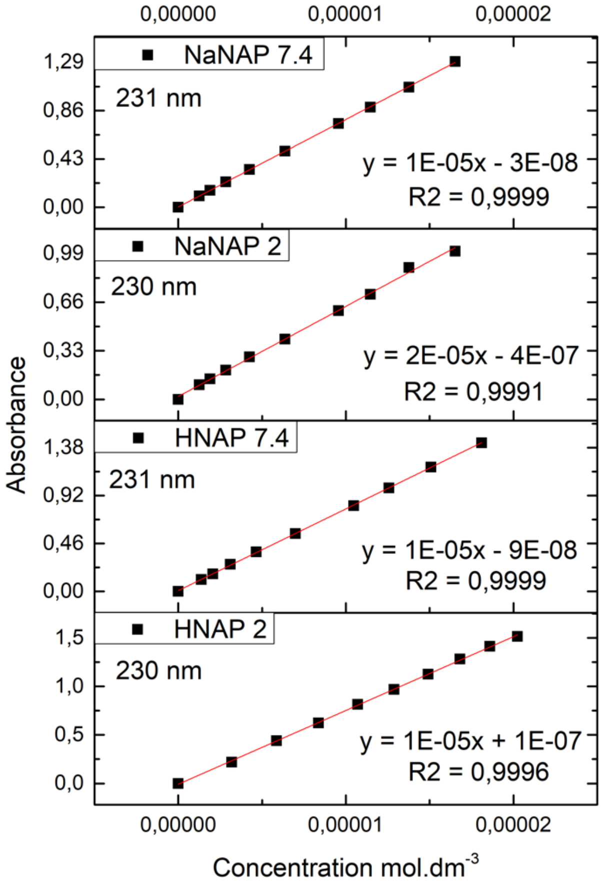

Figure S1. Calibration curves of naproxen sodium and naproxen acid at different *pH* obtained from UV measurements used for determination of total released amount of drug.

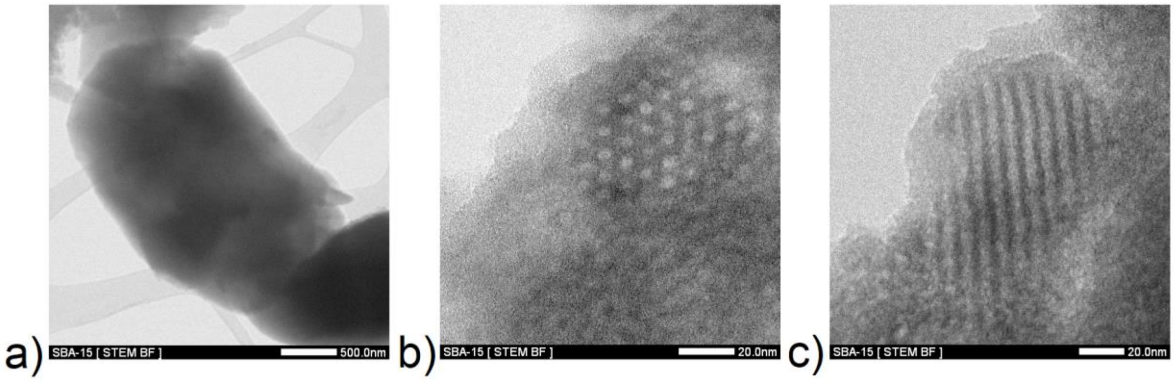

Figure S2. TEM images of prepared SBA-15 material: a) a shape of particles and a view along b) alo.,ng and c) parallel direction to the pores.

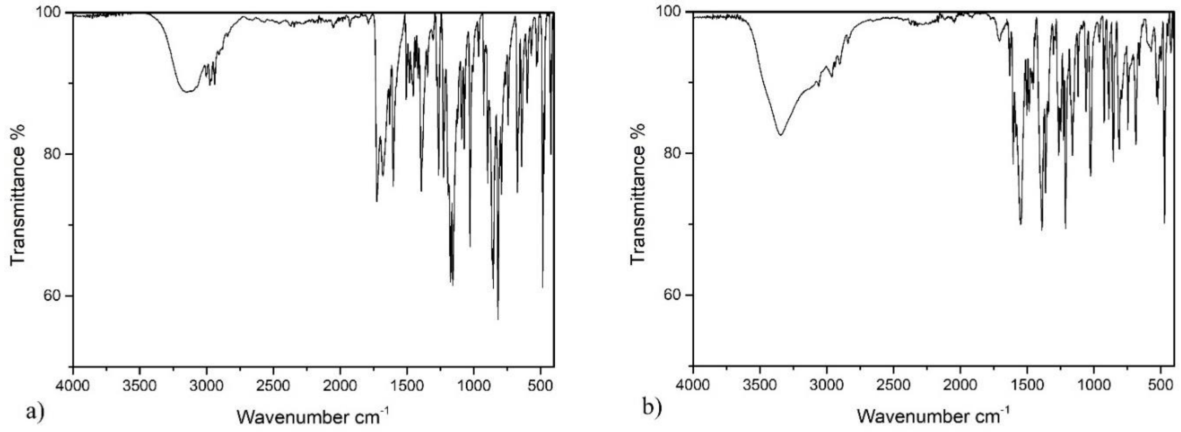

Figure S3 panel labels: a) b)

Figure S3. IR spectra of a) naproxen acid and b) naproxen sodium.

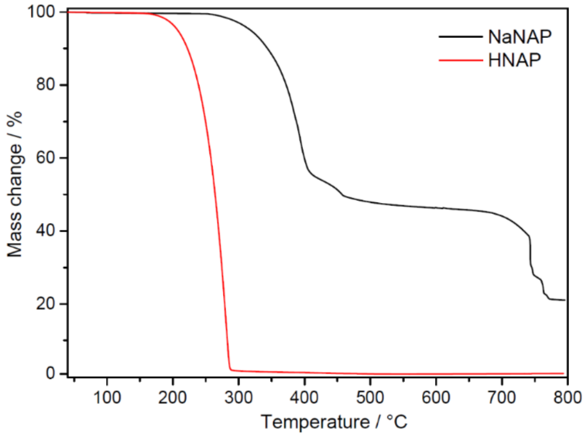

Figure S4. TG curves of pure drugs: naproxen sodium and naproxen acid measured in an air atmosphere.
